# Validation of an in vivo transit dosimetry algorithm using Monte Carlo simulations and ionization chamber measurements

**DOI:** 10.1002/acm2.14187

**Published:** 2023-10-27

**Authors:** David Sánchez‐Artuñedo, Savannah Pié‐Padró, Marcelino Hermida‐López, Maria Amor Duch‐Guillén, Mercè Beltran‐Vilagrasa

**Affiliations:** ^1^ Servei de Física i Protecció Radiològica Hospital Universitari Vall d'Hebron Barcelona Spain; ^2^ Institut de Tècniques Energètiques Universitat Politècnica de Catalunya Barcelona Spain

**Keywords:** Monte Carlo, PerFRACTION, PRIMO, transit dosimetry, validation

## Abstract

**Purpose:**

Transit dosimetry is a safety tool based on the transit images acquired during treatment. Forward‐projection transit dosimetry software, as PerFRACTION, compares the transit images acquired with an expected image calculated from the DICOM plan, the CT, and the structure set. This work aims to validate PerFRACTION expected transit dose using PRIMO Monte Carlo simulations and ionization chamber measurements, and propose a methodology based on MPPG5a report.

**Methods:**

The validation process was divided into three groups of tests according to MPPG5a: basic dose validation, IMRT dose validation, and heterogeneity correction validation. For the basic dose validation, the fields used were the nine fields needed to calibrate PerFRACTION and three jaws‐defined. For the IMRT dose validation, seven sweeping gaps fields, the MLC transmission and 29 IMRT fields from 10 breast treatment plans were measured. For the heterogeneity validation, the transit dose of these fields was studied using three phantoms: 10 , 30 , and a 3 cm cork slab placed between 10 cm of solid water. The PerFRACTION expected doses were compared with PRIMO Monte Carlo simulation results and ionization chamber measurements.

**Results:**

Using the 10 cm solid water phantom, for the basic validation fields, the root mean square (RMS) of the difference between PerFRACTION and PRIMO simulations was 0.6%. In the IMRT fields, the RMS of the difference was 1.2%. When comparing respect ionization chamber measurements, the RMS of the difference was 1.0% both for the basic and the IMRT validation. The average passing rate with a γ(2%/2 mm, TH = 20%) criterion between PRIMO dose distribution and PerFRACTION expected dose was 96.0% ± 5.8%.

**Conclusion:**

We validated PerFRACTION calculated transit dose with PRIMO Monte Carlo and ionization chamber measurements adapting the methodology of the MMPG5a report. The methodology presented can be applied to validate other forward‐projection transit dosimetry software.

## 1 INTRODUCTION

In vivo dosimetry is a quality assurance and safety tool that allows detection of deviations between delivered and planned dose, by means of dose measurements performed during the radiotherapy treatment administration.^1^ Initially, the most common practice was placing point detectors on the patient surface. Nevertheless, this method is time‐consuming^2^ and uncertainties related to the detector positioning and dose‐rate response reduce their utility in modulated plans.^3^ To go beyond the bounds of point dosimeter limitations, the use of transit dosimetry is spreading.^2^, ^4^, ^5^, ^6^, ^7^, ^8^, ^9^ Transit dosimetry is based on the transit images formed by the radiation reaching the electronic portal imaging device (EPID), through the patient, during field irradiation.

Early works on transit dosimetry^10^ used EPID images to manually calculate the delivered dose at a specific point in the planning CT. The delivered dose obtained from the transit images was then compared to the planning point dose value. Nowadays, several commercial systems are available that implement automated in vivo dosimetry based on EPID images.^2^ Some examples are Adaptivo (Standard Imaging, Wisconsin, USA), Dosimetry Check (currently distributed by LAP, Texas, USA), and the PerFRACTION software (Sun Nuclear Corporation, Melbourne, USA). Transit dosimetry software can be categorized into back‐projection and forward‐projection.^11^ In the back‐projection approach, as Dosimetry Check, a 3D dose distribution is calculated, in the planning CT, from the transit image and compared with the planned dose distribution. In the forward‐projection method, the transit image obtained is directly compared with an expected transit image calculated from the treatment plan and the planning CT.

The SunCHECK platform (Sun Nuclear Corporation, Melbourne, USA) includes an independent calculation software (DoseCHECK), and PerFRACTION, a forward‐projection software. PerFRACTION performs in vivo monitoring using the log‐files and the EPID images.

With the log‐files, the dose is calculated on the planning CT or the cone beam CT (CBCT) using a collapsed‐cone convolution/superposition algorithm.^12^, ^13^, ^14^ Then, the PerFRACTION calculated dose is automatically compared with the planned dose distribution. The comparison is reported as point difference at the isocenter, or a selected point by the user, a gamma analysis^15^ for the targets and the OARs selected and a 3D gamma analysis in the whole CT or CBCT.

Daily EPID transit images can be compared to a baseline image or to an expected transit dose calculated from the treatment plan. Baseline images are transit images obtained in previous treatment fractions. To compare the transit image to an expected transit dose, it is a prerequisite to calibrate PerFRACTION. The calibration process consists in the acquisition of the transit images of several static fields through solid water phantoms of different thicknesses. This calibration is applied to the transit images to convert them to a dose distribution calculated under the same conditions as the expected transit dose distribution.

The gold standard in the commissioning of software‐calculated doses in radiotherapy is the comparison of the calculated dose with the absorbed dose determined with dosimeters at the same conditions. To cover different treatment scenarios, such comparisons are carried out under different set‐ups. For example, the MPPG5a report^16^ presents a complete set of tests based on calculated dose comparison with experimental measures for the validation of treatment planning systems (TPS). The AAPM published in 2021 the TG‐219 report^17^ on the acceptance and commissioning of independent dose calculation systems, recommending the validation of the dose calculated with experimental measurements of the absorbed dose. Although transit dosimetry software is neither a TPS nor an independent dose calculation software, the user in the absence of concrete guidelines could try to adapt the tests presented in the aforementioned reports to perform the commissioning. In the back‐projection approach, the calculated dose and the measurements are performed at the patient level. Therefore, the measurement conditions presented in MPPG5a can be easily reproduced. Esposito et al.^18^ followed this approach to validate the transit dosimetry software Dosimetry Check, currently integrated in RadCalc (LAP), using slab phantoms with different densities (air, bone, and tissue) and anthropomorphic phantoms. Measurements were carried out with GafChromic EBT3 film.

In the case of a forward‐projection transit dosimetry software, however, the tests presented in the MMPG5a report cannot be easily performed, as the determination of absorbed dose must be made at the EPID level through the patient. Therefore, it is more difficult to validate a forward‐projection as existing guidelines assume that dose deposition is within the patient, a setup which can be easily mimicked with phantoms. In addition, it is important to consider how each software models the EPID. For example, the PerFRACTION algorithm internally replaces the EPID by a water phantom centered at the EPID level. This geometry − a water phantom placed at large source‐surface distance behind the external contour of the patient − excludes the use of the TPS to compare the dose calculated by both systems.

Therefore, with forward‐projection software, users often choose ways to validate the software that do not involve a determination of the absorbed dose under transit conditions. One option is to validate the sensitivity and specificity of these systems by testing them under known errors.^19^ Following this path, Zhuang et al.^20^ determined that PerFRACTION is sensitive to output variations of 0.2% and to 1 mm setup errors. Another option is to accumulate experience and to observe if there is a correlation between differences detected by the system and real deviations in treatment. In this case, Bossuyt et al.^21^ shared a 3‐year experience using PerFRACTION in clinical practice for several treatment sites. They reported different examples of failing fractions related to deviations in treatment delivery. Both approaches are reported in the recently published report of the Task Group 307^22^ on the use of EPID for transit dosimetry.

None of these procedures, however, allow the user to directly validate the expected absorbed dose calculated by the system. In such conditions, in which it is difficult to perform absorbed dose measurements, Monte Carlo codes for the simulation of radiation transport is considered as the gold standard.^23^ PRIMO^24^ is a Monte Carlo simulation software, freely downloadable from https://www.primoproject.net/. PRIMO performs simulations of Varian Clinac and TrueBeam linacs and estimates absorbed dose distributions in slab phantoms or CT image sets.

This work aims to validate the forward‐projection transit dosimetry algorithm of the PerFRACTION software (v. 2.10.0) with PRIMO Monte Carlo simulations and ionization chamber measurements.

## 2 MATERIAL AND METHODS

The PerFRACTION software requires an EPID calibration according to the linac, the MLC, and the EPID model, following manufacturer guidelines. In the specific case of a Varian TrueBeam STx v.2.7 (Varian Medical Systems, Palo Alto, California, USA) linac equipped with an HD‐MLC and a DMI EPID, the user needs to acquire the transit images of nine asymmetric fields defined with MLC and jaws through three attenuating phantoms. Specifically, the attenuation phantoms are composed of 30 cm thick solid water, 10 cm thick of solid water, and no‐attenuation phantoms (fields are delivered into air).

### 2.1 Validation fields

The validation protocol for the PerFRACTION transit dosimetry algorithm proposed in this work was designed to tailor the tests recommended in MMPG5a, as feasible, to the transit dosimetry conditions. The process was divided into three groups of tests according to MPPG5a^16^: basic dose validation, IMRT dose validation, and heterogeneity correction validation. A flow chart describing the validation process can be found in the supplementary material.

The fields used for the basic dose validation were the same nine asymmetric fields needed to calibrate PerFRACTION and three jaw‐defined square fields (6 × 6, 10 × 10, and 20 × 20 cm^2^). The dose distributions from the calibration fields were calculated with 50 monitor units (MU) according to the manufacturer protocol, while the rest of the static fields were calculated with 100 MU.

For the IMRT dose validation, first, we analyzed MLC characterization using seven sweeping‐gap fields (2, 4, 6, 10, 14, 16, and 20 mm) and a closed MLC field to measure MLC transmission. In both cases the jaws were set to a 10 × 10 cm^2^ field. We also measured two static MLC‐defined fields: 2 × 2 and 3 × 3 cm^2^ (in both cases jaws were set at 3.2 × 3.2 cm^2^). To test IMRT fields from clinical plans, 29 sliding‐window IMRT fields from 10 breast treatment plans were chosen. The prescription dose of the treatment plans was 40.5 Gy to the breast and 48 Gy to the simultaneous integrated boost, if needed. The number of beams per plan ranged from 2 to 5. In all cases, one of the fields corresponded to the inner tangential field and other to the outer tangential field. In those fields, the photon fluence was extended outside the body contour with the skin flash tool included in Eclipse. The angle of the other beams in the original plans was set according to the complexity of the treatment volumes and the geometry of the patient. A copy of the treatment plan was created and for every field studied, the gantry angle was re‐set at 0 degrees (Varian IEC scale).

To perform the heterogeneity correction validation, we used solid water slabs as attenuation phantom with the addition of a cork slab placed between solid water. We used cork, as according to Chang et al.,^25^ is a good lung substitute material. In total, the transit dose of the basic and the IMRT fields was studied on three solid water phantoms of varying thicknesses: 10 cm of solid water, 30 cm of solid water, and a 3 cm cork slab placed between two 10 cm slabs of solid water. Lateral and longitudinal dimensions of the phantom slabs were 30 × 30 cm^2^. Source‐surface distance (SSD) was set to 95 cm for the 10 cm and the 10 cm + cork phantom, and to 75 cm for the 30 cm phantom. Phantoms were virtually modelled in the TPS according to their physical dimensions and density.

The dose distributions from the calibration fields were calculated with 6 MV photons in Eclipse treatment planning system (Varian Medical Systems) with the analytical anisotropic algorithm (AAA) v. 15.6.4 using a 2.5 mm grid. The selected treatment unit was a TrueBeam STx linac equipped with an HD120 MLC.

### 2.2 Tolerances

The MPPG5a reports different tolerances depending on the tests analyzed. Although the tolerances are intended for TPS validation, in this work, they will be adopted as the accuracy target. In the case of the basic validation tests, it recommends a dose difference of 2% in the high dose region. Differences in the penumbra region of the fields should be evaluated using the distance to agreement with a tolerance of three mm. For the heterogeneity correction validation it recommends a 3% tolerance based on previous works such as Carrasco et al.^26^ and the IAEA TRS‐430 report.^27^ For IMRT validation, it recommends a tolerance of 2%, with 1.5% being preferable, for the average difference between ionization chamber measurements and doses calculated by the TPS. MPPG5a proposes the use of 2%/2 mm gamma criterion for the comparison of dose distribution. According to Opp et al.,^28^ with this criterion, easily correctable differences may appear, which could be unnoticed with a lax criterion such as 3%/3 mm. Although MPPG5a proposes a “no pass rate tolerance,” in this work, a 90% passing rate was used as evaluation criterion based on patient‐specific quality assurance protocols.^29^, ^30^, ^31^

### 2.3 PerFRACTION expected dose

The DICOM files corresponding to the plan, the structures, and the dose distribution were exported to PerFRACTION for all validation tests to calculate the expected dose. We define the field expected dose as the dose distribution in the middle plane of a 50 × 50 × 4.9 cm^3^ water phantom centered at the EPID plane, resulting from the field delivery through the patient. To calculate the expected doses, the PerFRACTION software uses its own beam model based on a type‐B collapsed‐cone algorithm.^32^ A local bespoke beam model was used. Each field‐phantom combination was delivered in the TrueBeam STx. During delivery, transit images were acquired with the EPID placed at 150 cm from the source using the dosimetry mode. Dosimetry mode calibration requires a dark field, a flood field, a beam profile correction and to define the calibration units (CU) normalization. One CU signal at central axis is produced when the reference field (100 monitor units and 10 × 10 cm^2^ field size) is delivered.

PerFRACTION converted the transit images to an absorbed dose distribution, namely, delivered dose, in the same phantom plane than the expected dose.

For each transit image, the PerFRACTION expected dose in the central point of the EPID was obtained for comparison with measurements and with the PRIMO‐estimated dose value. We prioritized that the expected dose value corresponded to a homogeneous and representative region, thus in some IMRT fields not all the points were obtained from the EPID central point.

### 2.4 PRIMO Monte Carlo simulations

We used PRIMO (v. 0.3.64.1814) to simulate the dose distribution in the same dose plane as the expected dose calculated by PerFRACTION. PRIMO allows performing the simulations using two Monte Carlo engines: PENELOPE^33^ and the Dose Planning Method (DPM).^34^, ^35^ We chose the DPM engine for this work, as it a fast Monte Carlo code designed for radiotherapy problems. Previous works have validated the PRIMO software simulations of Varian linacs. Brualla et al.^36^ compared the spectra calculated using PRIMO with other Monte Carlo systems. Moreover, Brualla et al.^37^ reviewed the different Monte Carlo systems used for simulating RT plans, including PRIMO. Hermida‐López et al.^38^ benchmarked PRIMO simulations against the IROC‐Houston external beam audit data, which include 11 TrueBeam linacs. Esposito et al.^39^ validated the Millennium 120 MLC for the simulation of IMRT plans and Paganini et al.^40^ validated the HD120 MLC for VMAT plans. Other applications of PRIMO included the use of PRIMO as an independent tool for the beam commissioning of a 6 MV Varian Truebeam STx,^41^ simulation of out‐of‐field dose,^42^ simulation of conical collimators for stereotactic radiosurgery,^43^ and the use of PRIMO as an independent dose verification system for radiosurgery HyperArc plans.^44^

Table 1 summarizes the simulations details following the scheme proposed by the AAPM Task Group 268 report RECORDS.^45^

**TABLE 1:** Details of the simulations in this work, following the scheme proposed by the AAPM Task Group 268 report RECORDS.^45^

Item	Description	References
Monte Carlo code	PRIMO v. 0.3.64.1814, with DPM simulation engine.	^24^, ^38^, ^41^, ^46^, ^47^
Hardware	Processor: Intel Xeon CPU E5‐2630 v2 @2.60 GHz (24 CPUs) , with 32 GB of RAM.	
Simulation time	10x10 cm^2^ field in a 30.5 × 30.5 × 30 cm^3^ water phantom with 0.5 × 0.5 × 0.2 cm^3^ bins at SSD = 90 cm (reference conditions): 2.4 h.	
	10 × 10 cm^2^ field in 50.5 × 50.5 × 57.45 cm^3^ phantom (slab materials: solid water, air and water) with 0.5 × 0.5 × 0.05 cm^3^ bins at SSD = 95 cm (PerFRACTION conditions for the 10 cm water solid phantom): 6.7 h.	
Geometry	Slabs phantoms: Slabs corresponding to the attenuation phantom (i.e., 10 cm of solid water), couch: 0.5 cm water slab, air slab, 4.9 cm water slab (representing EPID)	
Materials	PRIMO default materials with the exception of cork, for which the density was changed to 0.25 g/cm^3^ according to vendor specifications.	
Source	Varian TrueBeam 6 MV phase‐space file (PSF, v.2, Feb.27 2013) in IAEA format.	
Physics and transports	Cross sections: PENELOPE 2011 and DPM (Klein‐Nishina for Compton scattering, Møller for electron inelastic collisions)	^33^, ^34^, ^35^, ^48^, ^49^, ^50^
	PRIMO default transport parameters: Woodcock tracking technique.	^51^, ^52^, ^53^
	Variance reduction technique: simple particle splitting in the voxelized geometry with a splitting factor of 170.	
Scoring	Absorbed dose at the EPID plane (middle plane of the 4.9 cm water slab). Bin size 0.5 × 0.5 × 0.05 cm^3^, except for the 2 × 2 and 3 × 3 cm^2^ fields (bin size of 0.1 × 0.1 × 0.05 cm^3^)	
	4.9 × 10^10^ histories per simulation.	^33^
	Voxel‐by‐voxel statistical uncertainty reported with *k* = 2.	
Analysis	No post‐processing of the simulation results was applied.	

PRIMO divides the dose simulation into three stages: the upper part of the linac, from the exit of the accelerating waveguide to a plane right above the secondary collimator jaws (stage s1). The stage s2 simulates the jaws and the MLC and thus is field‐dependent. The stage s3 allows the simulation of the dose deposition on a CT or a slab phantom. Instead of simulating the stage s1 we used the TrueBeam phase‐space file (PSF) provided by Varian for 6 MV , with 4.9 × 10^10^ histories . This PSF was used as particle source for all the s2 + s3 stages simulations. For the s2 stage either, we defined a jaw position in PRIMO or we imported a DICOM plan. In the specific case of sweeping‐gap fields we increased the number of control points by a factor of 10 using the PRIMO DICOM import plan options, to ensure that the simulation represented a continuous MLC movement as in the beam delivery.

In the s3 stage, we defined slab phantoms consisting of four parts. The first part represents the material and geometry of one of the three attenuation phantoms defined previously: 10 cm of solid water, 5 cm of solid water + 3 cm of cork + 5 cm of solid water, or 30 cm of solid water. The second part was the treatment couch, which was replaced by a slab with a thickness of 0.5 cm of water for its equivalent attenuation according to Varian and our experimental measurements. The third part was an air slab, and the final part was a slab of 4.9 cm of water representing the EPID, to match PerFRACTION expected dose calculation conditions. The air slab thickness was calculated so that middle plane of the water slab representing the EPID, that is, at 2.45 cm from the surface, was at a distance of 50 cm from the isocenter and, therefore, at a distance of 150 cm from the source. As an example, when simulating the 10 cm solid water phantom placed over the couch at a SSD of 95 cm, the slabs used were as follows: 10 cm of solid water, 0.5 cm of water, 42.05 cm of air, and 4.9 cm of water.

The size of the phantom set in PRIMO was 50.5 × 50.5 cm^2^ in the lateral and longitudinal directions to match PerFRACTION calculation conditions. The bin size was 0.5 cm in these directions, and 0.05 cm in the vertical direction, except for the small MLC‐defined fields in which the phantom size was 10.1 × 10.1 cm^2^ with a bin size of 0.1 cm × 0.1 cm × 0.05 cm. An even number of bins was chosen in the lateral and longitudinal directions to ensure that the central axis of the beam (CAX) was centered in a bin.

All simulations were carried out with simple particle splitting at the phantom to reduce variance, with a splitting factor of 170, which was determined empirically. We did not apply any post‐processing to the simulated dose distributions.

From every simulation, we obtained the point dose value and its statistical uncertainty at the depth of 2.45 cm of the water‐EPID at the same position of the PerFRACTION expected dose value. The simulated 2D‐dose distribution at the depth of 2.45 cm was exported from PRIMO and compared with the PerFRACTION expected dose distribution using the OmniPro software v.1.7.2001 (IBA). We evaluated the passing rate, defined as the percentage of points with gamma index value below 1, with three global gamma criteria: γ(1%/1 mm, threshold (TH) = 20%), γ(2%/2 mm, TH = 20%), and γ(3%/3 mm, TH = 20%). We used a 20% threshold to exclude from the analysis the low‐dose region due to uncertainties in the dose conversion from EPID images.^22^ No interpolation was applied to the PRIMO‐estimated dose.

### 2.5 Ionization chamber measurements

**FIGURE 1:**
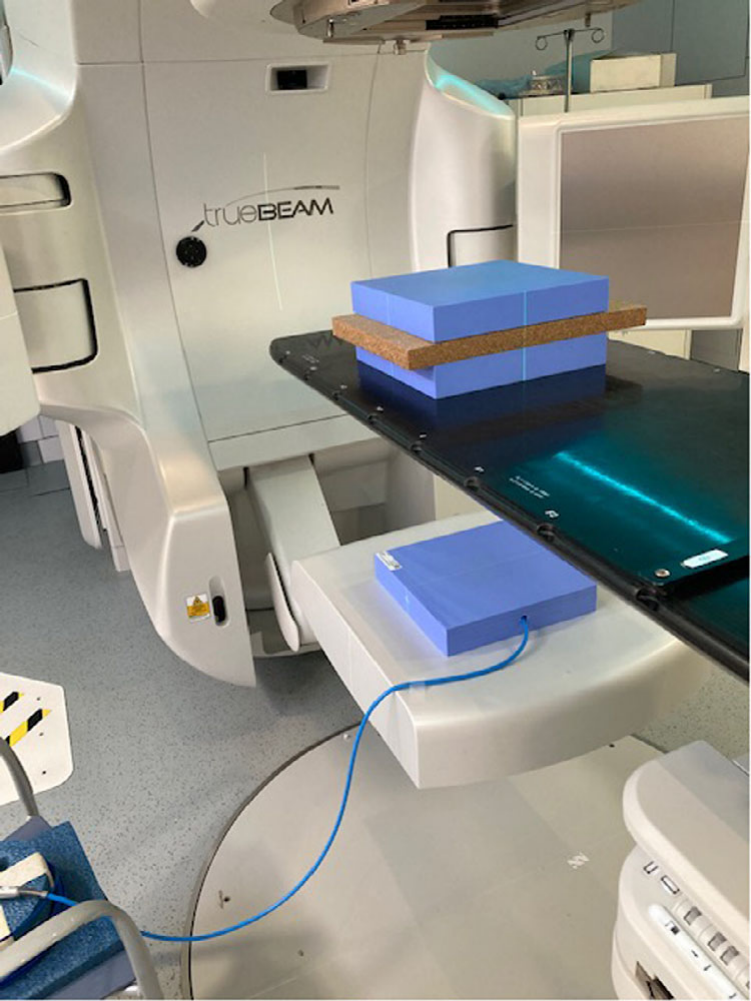
Set‐up used to perform ionization chamber measurements.

Figure 1 shows an example of the set‐up used to carry out the ionization chamber measurements. A solid water phantom (Solid Water HE, Sun Nuclear) was placed on the EPID cover. The bottom part of the measurement phantom consisted on a 2 cm solid water slab (backscatter slab) below a 2 cm solid water slab with a hollow insertion for the ionization chamber. The ionization chamber slab is marked with a cross on its surface to help placing the phantom aligned with the beam axes using the in‐room lasers. When the ionization chamber is inserted in the slab, the effective measurement point of the chamber is centered in the CAX, and located at the middle plane of the slab. Once the phantom was centered and the chamber placed, another 1.5 cm slab of solid water was added on top as build‐up. Therefore, the measurement phantom consisted on a solid water phantom of 30 × 30 cm^2^ and 5.5 cm thick with a chamber placed at a depth of 2.5 cm. The vertical axis of the EPID was adjusted until the ionization chamber effective measurement point was placed at a distance of 150 cm from the source.

The attenuation phantoms used to perform PerFRACTION measurements were placed on the treatment couch and the SSD was adjusted to 95 cm for phantoms with 10 cm of solid water, and to 75 cm for 30 cm solid water phantom. Figure 1 shows the set‐up used to carry out the ionization chamber measurements. The phantom was moved laterally, if necessary, to reproduce the PerFRACTION representative points.

IMRT fields, 2 × 2, and 3 × 3 cm^2^ MLC‐defined fields were measured with a PTW 31016 PinPoint3D ionization chamber (sensitive volume 0.016 cm^3^). The rest of the fields were measured using a PTW 30013 Farmer‐type ionization chamber (sensitive volume 0.6 cm^3^). Both chambers were used in conjunction with a PTW Unidos E electrometer.

The PerFRACTION expected dose, the PRIMO simulation results, and the ionization chamber measurements were compared.

### 2.6 Uncertainties

The following sources of uncertainty were considered:

- PerFRACTION uncertainties: i) general collapsed‐cone algorithm uncertainties, ii) uncertainty of the reference dose used for the calibration of the software, and iii) resolution of the displayed dose value.
- Simulation uncertainties: i) statistical uncertainty from the PSF calibration, ii) uncertainty of the reference dose used for the PSF calibration, iii) uncertainties from the linac model included in PRIMO, iv) uncertainties due to variations in cross‐sections, v) uncertainty related to the use of DPM as simulation engine instead of PENELOPE, and v) statistical uncertainties of the estimated dose distributions.
- Ionization chamber measurements: i) detector calibration coefficient, ii) quality factor *k_Q_*, iii) experimental set‐up, iv) reading correction for influence quantities as pressure, temperature, polarity and recombination; v) EPID‐backscatter, and vi) linac output repeatability.

## 3 RESULTS

### 3.1 Basic dose validation

**FIGURE 2:**
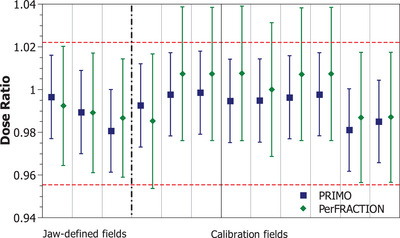
Results of the dose ratio of PerFRACTION expected dose and PRIMO‐estimated dose respect the ionization chamber measurements for the basic validation fields delivered in the 30 × 30 × 10 cm^3^ phantom. Uncertainty bars are plotted with a coverage factor of *k* = 2. The dashed horizontal red line represents the ionization chamber measurements uncertainty with a coverage factor of *k* = 2.Vertical dot‐dashed line separates jaw‐defined fields from calibration fields.

Figure 2 shows the dose ratio of PerFRACTION expected dose and PRIMO estimated dose respect to the ionization chamber measurements under transit dosimetry conditions in the 30 × 30 × 10 cm^3^ phantom. The first three fields correspond to the three jaw‐defined fields (6 × 6, 10 × 10, and 20 × 20 cm^2^) fields and the other nine are the asymmetric fields needed to calibrate PerFRACTION.

In this phantom, the root mean square (RMS) of the differences between PerFRACTION expected dose and the dose determined with ionization chamber measurement was 1.0% both for the jaw‐defined fields [−0.9%, 1.2%], and for the asymmetric fields [−1.7%, 0.8%]. When comparing PerFRACTION expected doses against PRIMO estimated doses, the RMS of the differences were 0.4% [−0.4%, 0.6%] for the jaw‐defined fields and 0.8% [−0.4%, 1.3%] for the asymmetric fields.

Table 2 shows the gamma passing rate obtained from the comparison of PRIMO and PerFRACTION expected doses using the different gamma criteria studied. The 6 × 6 cm^2^ field and three calibration fields had passing rates below 90% when using a γ(2%/2 mm, TH = 20%) criteria. With a γ(3%/3 mm, TH = 20%) criteria, all fields used for the basic dose validation had a passing rate above 95%.

**TABLE 2:** Results of the gamma comparison of PerFRACTION expected dose distribution and PRIMO‐estimated dose distribution for different fields used for the basic validation delivered in the 30 × 30 × 10 cm^3^ phantom.

Field	Passing rate γ(1%/1 mm, TH = 20%)	Passing rate γ(2%/2 mm, TH = 20%)	Passing rate γ(3%/3 mm, TH = 20%)
6 × 6 cm^2^	54.8%	75.6%	99.7%
10 × 10 cm^2^	49.5%	90.3%	100.0%
20 × 20 cm^2^	87.7%	93.8%	96.8%
Calibration fields	59.1% ± 24.8%	92.7% ± 6.3%	99.0% ± 1.2%

The results of the nine calibration fields are presented as the average results and the standard deviation for simplicity.

### 3.2 IMRT dose validation

The PerFRACTION expected doses for the MLC‐defined fields, 2 × 2 and 3 × 3 cm^2^, presented a difference respect to measurements of 1.0% and 1.3%, respectively. When comparing with PRIMO estimated dose, this difference is 1.3% and 1.2%, respectively.

**FIGURE 3:**
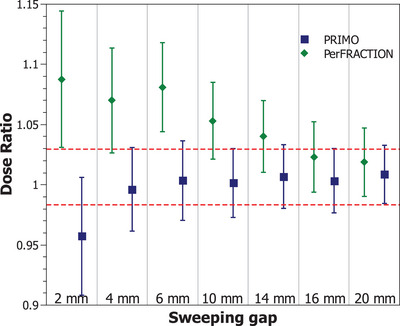
Results of the dose ratio of PerFRACTION expected dose and PRIMO‐estimated dose respect to the ionization chamber measurements for the sweeping‐gap fields delivered in the 30 × 30 × 10 cm^3^ phantom. Uncertainty bars are plotted with a coverage factor of *k* = 2. The dashed red line represents the ionization chamber measurements uncertainty.

Figure 3 shows the dose ratio of PerFRACTION expected dose and PRIMO estimated dose respect to the ionization chamber measurements in the 30 × 30 × 10 cm^3^ phantom for the sweeping‐gap fields. The RMS of the differences between PerFRACTION expected dose and the dose determined with ionization chamber measurement was 5.9 % [1.9%, 8.8 %]. When comparing PerFRACTION against PRIMO, the RMS of the differences was 7.0% [1.0%, 13.6%]. The measured transmission of the HD120 MLC was 1.12% ± 0.04% respect to the reference 10 × 10 cm^2^ field under transit conditions, while the PRIMO estimated transmission was 0.83% ± 0.07% and the PerFRACTION expected transmission was 1.11% ± 0.18%. Uncertainties reported with a coverage factor of *k* = 2.

**FIGURE 4:**
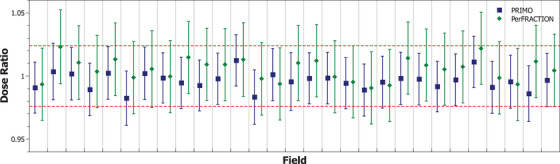
Results of the dose ratio of PerFRACTION expected dose and PRIMO‐estimated dose respect the ionization chamber measurements for the IMRT fields delivered in the 30 × 30 × 10 cm^3^ phantom. Uncertainty bars are plotted with a coverage factor of *k* = 2. The dashed red line represents the ionization chamber measurements uncertainty.

Figure 4 shows the corresponding results for the IMRT fields. The RMS of the differences between PerFRACTION expected dose and the dose determined with ionization chamber measurement was 1.0% [−0.9%, 2.3%]. When comparing PerFRACTION against PRIMO, the RMS of the differences was 1.2% [−0.7%, 2.6%].

Table 3 presents the gamma passing rate obtained from the comparison of PRIMO and PerFRACTION expected doses using the different gamma criteria studied. Only the MLC‐defined 2 × 2 cm^2^ field had passing rates below 90% when using a γ(2%/2 mm, TH = 20%) criterion. With a γ(3%/3 mm, TH = 20%) criterion, all fields used for the basic dose validation had a passing rate above 90%.

**TABLE 3:** Results of the gamma comparison of PerFRACTION expected dose distribution and PRIMO‐estimated dose distribution for different fields used for the IMRT validation delivered in the 30 × 30 × 10 cm^3^ phantom.

Field	Passing rate γ(1%/1 mm, TH = 20%)	Passing rate γ(2%/2 mm, TH = 20%)	Passing rate γ(3%/3 mm, TH = 20%)
MLC: 2 × 2 cm^2^	59.2%	79.2%	93.8%
MLC: 3 × 3 cm^2^	46.0%	94.7%	100.0%
IMRT fields	79.5% ± 7.5%	98.7% ± 1.6%	100% ± 0.1%

For simplicity, the results of the 29 IMRT fields are presented as the average results and the standard deviation.

### 3.3 Heterogeneity correction validation

**FIGURE 5:**
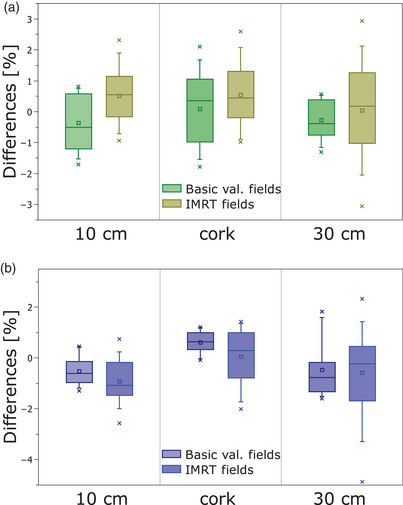
Results of the differences of PerFRACTION expected dose respect: (a) ionization chamber measurements and (b) PRIMO for different phantoms and validation fields. Cross symbols represents maximum and minimum value, whiskers 5^th^ and 95^th^ percentile, lines 25^th^, 50^th^, and 75^th^ percentile, and square symbol the mean value.

Figure 5a shows the results of the comparison of PerFRACTION expected dose respect to ionization chamber measurements for different attenuation phantoms. For the basic validation fields, the RMS of the differences were 1.0% [−1.7%, 1.3%] for the 10 cm phantom, 1.2% [−1.8%, 2.1%] for the cork phantom, and 1.2% [−1.3%, 2.9%] for the 30 cm phantom. For the IMRT fields, the RMS of the differences were 1.0% [−0.9%, 2.3%] for the 10 cm phantom, 1.0% [−1.0%, 2.6%] for the cork‐phantom, and 1.4 % [−3.0%, 2.9%] for the 30 cm phantom.

Figure 5b shows the results of the comparison of PerFRACTION expected dose with respect to PRIMO‐estimated doses. For the basic validation fields, the RMS of the differences were 0.8% [−0.5%, 1.3%] for the 10 cm phantom, 0.8% [−1.2%, 1.4%] for the cork phantom, and 1.5% [−1.8%, 2.9%] for the 30 cm phantom. For the IMRT fields, the RMS of the differences were 1.2% [−0.7%, 2.6%] for the 10 cm phantom, 1.0% [−1.4%, 2.0%] for the cork‐phantom, and 1.7% [−2.3%, 4.9%] for the 30 cm phantom.

Table 4 presents the gamma passing rates obtained from the comparison of PRIMO and PerFRACTION expected doses for different attenuation phantoms using a γ(2%/2 mm, TH = 20%) criteria.

**TABLE 4:** Passing rates of the gamma index comparison of PerFRACTION expected dose distribution and PRIMO‐estimated dose distribution for different fields and phantoms using a γ(2%/2 mm, TH = 20%) criteria.

Field	10 cm solid water phantom	10 cm solid water phantom+3 cm cork	30 cm solid water phantom
6 × 6 cm^2^	75.6%	89.1%	77.1%
10 × 10 cm^2^	90.3%	85.0%	88.7%
20 × 20 cm^2^	93.8%	93.8%	93.8%
Calibration fields	92.7% ± 6.3%	90.4% ± 11.3%	93.2% ± 5.2%
MLC: 2 × 2 cm^2^	79.2%	79.6%	75.5%
MLC: 3 × 3 cm^2^	94.7%	87.4%	92.9%
IMRT fields	98.7% ± 1.6%	98.9% ± 1.3%	92.3% ± 5.5%

The results of the nine calibration fields and the 29 IMRT fields are presented as average results with the standard deviation for simplicity.

### 3.4 Uncertainties

Table 5 summarizes the uncertainty evaluation for the experimental measurements, the PRIMO simulations, and the PerFRACTION transit calculated doses. The uncertainty of the detector calibration coefficient was taken from the calibration certificate of the ionization chambers, which were issued by the PTW Calibration Lab traceable to the German National Laboratory. The uncertainty related to the quality factor *k_Q_* was obtained from the IAEA TRS‐398 code of practice.^54^ The small differences between the measurement phantom geometry and the PerFRACTION/Monte Carlo set‐up in terms of backscatter and measurement depth are included in the experimental set‐up uncertainties. The statistical uncertainty of the simulations was reported as the minimum‐maximum interval. Maximum uncertainty, for every test type, was obtained using the attenuation phantom of 30 cm thick. Sempau et al.^35^ reported differences of less than 1% when comparing DPM with PENELOPE. The largest differences were obtained in air and in the build‐up region. However, in this work, the dose estimated with PRIMO was obtained in a water phantom and after the build‐up region. Other sources of uncertainty related to Monte Carlo simulations as variations in the linac geometry and the cross‐sections included in the code are difficult to determine. Nevertheless, an estimated of the combined effect (DPM, cross‐sections, and linac geometry) can be obtained from previous comparison with experimental measurement, as performed by Hermida‐López et al.^38^ The PerFRACTION uncertainty related to a general collapsedcone algorithm was also taken from the literature.^55^

**TABLE 5:** Uncertainty budget.

Source	Type	Uncertainty (%)	Total (%)
Ionization chamber measurements			
Detector calibration coefficient	B	1.1	
Quality factor *k_Q_* ^34^	B	1.0	
Experimental set‐up	A	1.9	
Reading correction for influence quantities as pressure, temperature, polarity and recombination	A	0.8	
EPID‐backscatter	A	0.4	
Linac output repeatability	A	0.4	
			2.6
Simulation uncertainties			
PSF calibration	A	0.3	
Reference dose used for PSF calibration	A	1.7	
Other sources of uncertainties: DPM,linac model, cross sections^34^	B	0.3	
Voxel‐based statistical uncertainties of the estimated dose distribution:	A		
Basic dose validation		0.7	1.9
IMRT fields		[0.8, 1.4]	[2.0, 2.3]
Sweeping‐gap fields		[1.6, 4.5]	[2.4, 4.9]
PerFRACTION uncertainties:			
Collapsed‐cone algorithm^48^	B	2	
Reference dose used for PSF calibration	A	1.7	
Resolution in the displayed dose value (0.5 mGy)	A		
Basic dose validation		[0.2, 0.4]	[2.8, 3.1]
IMRT fields		[0.2, 1.1]	[2.9, 3.0]
Sweeping‐gap fields		[1.0, 5.0]	[2.8, 5.7]

Values are reported with *k* = 2.

## 4 DISCUSSION

We validated PerFRACTION expected transit dose using ionization chamber measurements and Monte Carlo simulations. We chose to adapt the MPPG5a practice guideline^16^ on TPS validation. For the basic photon beam validation, the MPPG5a guideline proposes three tests (5.1 to 5.3). In these tests, the absorbed dose, the PDD, and off axis output factor for small and large field sizes must be checked under reference conditions. We adapted these tests to the PerFRACTION validation, by using the jaw‐defined fields for the basic validation. Of course, the PDD cannot be checked on a 2D‐dose distribution. To overcome this limitation, we compared the CAX dose calculated by PerFRACTION with the simulated and the experimental values. Moreover, we compared the PerFRACTION expected transit dose distribution with respect to PRIMO estimated dose distribution. The comparison was made for the three jaw‐defined fields and with three different attenuation phantoms to fulfill the MPPG5a 5.1 to 5.3 test requirements.

Tests 5.4 to 5.7 require the validation of different MLC‐shaped field: small and large field, off‐axis, and asymmetric field at minimal SSD. We adapted these tests by using the calibration fields and the 30 × 30 × 30 cm^3^ solid water phantom (with an SSD of 75 cm for the attenuation phantom). The purpose of using these calibration fields and the 30 × 30 × 30 and 30 × 30 × 10 cm^3^ solid water phantoms is twofold: 1) to comply with the MMPG5a test requirements, and 2) to validate the fields and conditions used to calibrate PerFRACTION.

Test 5.8, 10 × 10 cm^2^ field at oblique incidence, is not performable as is in transit dosimetry conditions, because oblique gantry incidence cannot be used as the EPID rotates in solidarity with the gantry and therefore a safe position for the experimental set‐up cannot be guaranteed.

We did not perform test 5.9 as we do not use wedges in clinical practice in this linac. MMPG5a propose a 2% dose difference and 3 mm distance‐to‐agreement tolerance for this test.

With a γ(2%/2 mm, TH = 20%) criterion four fields had passing rates below 90%. Nevertheless, with a γ(3%/3 mm, TH = 20%) criterion all fields used for the basic dose validation had a passing rate above 95%. However, taking into account that the uncertainties are greater under transit conditions (greater difficulty of positioning, lower deposited dose due to higher attenuation, etc.) using a tolerance of 3% for dose difference seems reasonable.

To perform the heterogeneity correction validation MPPG5a proposes two tests: test 6.1 to validate the CT‐density calibration, and test 6.2 to validate the heterogeneity correction measuring and calculating above and below the heterogeneity. Test 6.1 does not apply to transit dosimetry as the EPID is considered as water. In fact, the user will have already validated the CT‐density calibration previously when commissioning the independent calculation software and the dose deposition algorithm. This test is therefore outside the scope of this work. In the same sense, it is not possible to measure above and below the heterogeneity as in forward‐projection transit dosimetry the calculated dose is always at the EPID level. Therefore, the measurement is performed after the patient and the heterogeneity. However, we believe that the validation of any software should include the study of heterogeneities, especially lung, as they are present in all the chest clinical cases. To this purpose, we performed all the tests studied also in a phantom with a 3 cm cork slab, as a lung surrogate. Maximum mean differences between phantoms were of 1% and the passing rates obtained were very similar regardless the phantom used.

Therefore, the PerFRACTION (collapsed‐cone algorithm) heterogeneity correction results are within the expected values. This result is not surprising as this type of evaluations have been widely reported previously in the literature.^56^, ^57^, ^58^

For IMRT acquisition, the MMPG5a recommends following the manufacture recommendations for measuring intra‐leaf and inter‐leaf MLC transmission. To this purpose, we measure both MLC transmission and several sweeping‐gap fields. In the sweeping‐gaps, we obtained the biggest differences between PerFRACTION and both experimental measurements and PRIMO simulations. Although the results of the ionization chamber measurements and PRIMO simulations are consistent within the experimental uncertainty, the differences with PerFRACTION in these fields are approximately 5%. These differences may be caused by several reasons. On the one hand, the sweeping gaps, specially the smallest ones, are near‐closed fields. Thus, the transit doses are on the order of a few mGy and any difference is magnified if expressed as percentage. For example, in the 2 mm gap, the dose determined with the ionization chamber was 9.2 mGy, while the expected dose from PerFRACTION was 10 mGy. In addition, during the commissioning of PerFRACTION, the generic model provided by Sun Nuclear is fine‐tuned by the manufacturer using a set of representative treatment plans provided by the user. These plans must have been previously validated by the user with experimental measurements to ensure the dose calculated by the TPS is correct. In the tuning process, the PerFRACTION algorithm is adjusted to minimize the differences of the calculated dose with respect the TPS for these plans. In our center, we tuned the MLC parameters in the TPS to match the experimental results of clinical IMRT and VMAT plans. Therefore, when adjusting PerFRACTION to the TPS, which has been adjusted to clinical plans and not to experimental measurements of sweeping‐gap fields, these differences are likely to arise.

Once the MLC was validated, MPPG5a proposes the validation of small MLC‐shaped fields (tests 7.1 and 7.2). In this case, we obtained similar results than in the jaw‐defined regular‐size fields.

To finish the validation process, we carried out measurements and simulations on 29 breast IMRT plans (test 7.4). The RMS of the dose difference with ionization chamber measurement was 1.0% and with PRIMO 1.2%. The average gamma passing rate with a γ(2%/2 mm, TH = 20%) criteria over the three phantoms studied was 94.4% ± 5.4%. Hence, the differences obtained in the sweeping gap fields do not have an implication on the clinical IMRT fields. We chose breast plans, as they are usually contain large fields, with fluence gradient and, depending on the case, they can be quite modulated. We obtain higher passing rates for the IMRT fields than for the calibration or the square fields. In the second group of fields, differences were observed in the penumbra region. However, for the IMRT fields, no differences were found in the penumbra region, and the failing points were located in‐field. As discussed previously, one possible explanation is the model fine‐tuning by the manufacturer using modulated clinical plans.

We relied on ionization chamber point measurements as we had to discard 2D detectors as radiochromic film detector arrays. According to Table 1 of the Report of AAPM Task Group 235,^59^ the useful dose range of EBT3 film is above 10 mGy, which is within the range of transit dose. So, when performing a film measurement under transit condition two main problems arise: 1) being out of specifications or at the lower dose range, thus increasing uncertainty, and 2) the increase of film noise due to the low signal of the transit dose. The use of 2D‐array detectors is also not possible, as it is not feasible to place them above the EPID and they have a predetermined geometry so the measuring detector cannot be placed at the same depth and with the same amount of backscatter material to match transit conditions.

Monte Carlo codes do not use dose deposition models; instead, the effective cross sections of the materials are used to reproduce the interaction with the radiation. Therefore, it can be assumed that if a Monte Carlo code agrees with measurements under standard conditions, it will agree also with experimental measurement when used in transit conditions, provided an accurate description of the geometry and materials of the system. The only obstacle is that under transit conditions fewer particles will reach the measurement plane and thus it will increase the voxel uncertainty. This effect can be observed when comparing the statistical uncertainty of the simulations when using the 10 cm and the 30 cm attenuation phantom. For the IMRT fields, with the first phantom the statistical uncertainty range was [0.8%, 1.4%] and with the thicker phantom was [1.2%, 2.0%]. Excluding the sweeping‐gap fields, the average uncertainty obtained in the PRIMO‐simulated dose values used to compare with ionization chamber measurements was 1% (*k* = 2). This value is higher than the reported values in the literature^38^, ^41^ for simulations on phantoms with this code. As stated before, this is because under transit dosimetry conditions less dose deposition is produced in the measurement plane. In this work, the EPID was replaced by a homogeneous water phantom to match PerFRACTION expected dose calculation conditions. However, the use of Monte Carlo simulations to validate EPID portal dose has been reported previously on the literature. Early works had characterized energy deposition and quantum noise in an EPID^60^, ^61^, ^62^ and studied the effect of scattered radiation on the formed signal. ^63^, ^64^, ^65^ Siebers et al.^66^ reproduced the geometry of an as500 EPID (Varian Medical Systems, Palo Alto, California, USA) and simulated the energy deposition of different validation fields using the EGS4^67^ Monte Carlo code. Differences between experimental measurements and Monte Carlo simulations were less than 1% for different static fields. Using a 2%/2 mm gamma criterion, passing rates over 98% were obtained for MLC sweeping gaps and 99% for an IMRT test field.

We decided not to interpolate the PRIMO dose distribution obtained since the AAPM TG‐218 report^29^ recommends interpolating the expected dose distribution (PerFRACTION) only if the spatial resolution is bigger than a third of the DTA.^68^ The spatial resolution of PerFRACTION calculated transit dose is 0.6 mm, which is lower than one‐third of the 2 mm and the 3 mm DTA. Moreover, the OmniPro manual advices that in case of different spatial resolution the reference distribution (PRIMO) should be the one with the coarser resolution. This condition is met according PRIMO spatial resolution is 5 mm.

The main limitation of the methodology presented in this work is that it allows validating fields with gantry only at 0 degrees. Despite this limitation, we obtained the dose difference values for fixed fields both static and dynamic MLC. In both situations, most of the analyzed fields present a dose difference of less than 3%. When a user has to commission a transit dosimetry system, one of the most complicated parts is to establish the different metrics for the analysis of the transit images. A good approach can be to accumulate experience and set the metrics as a function of location as presented by Bossuyt et al.^21^ The gamma criterion with the smallest dose difference they use is a γ(3%/3 mm, TH = 20%) in the case of head and neck, and brain treatments. It seems reasonable to users that the most restrictive metric is used for the best immobilized location. We do not recommend using lower dose difference values than 3%, as it is of the order of the differences and uncertainties obtained in this work. Based on AAMP Task Group 307^22^ report, users should stablish passing rate tolerance based on literature and their own commissioning process.

This paper proposes a methodology to validate forward‐projection transit dosimetry software with both experimental measurements and Monte Carlo simulations. Esposito et al.^18^ presented the validation of a back‐projection software with slab and anthropomorphic phantoms. Previous works^69^, ^70^ also had validated the PerFRACTION collapsed‐cone convolution/superposition algorithm with experimental measurements performed with ionization chambers and detector arrays. However, in all these works, the calculated dose and the measurements were performed at the patient level. In a forward‐projection transit dosimetry software, the transit image is calculated under different conditions, as it is obtained after the patient at the EPID level and not in the patient. At the present time, the validation of the expected transit dose of a forward‐projection software is not reported in the literature.

The method presented for the validation of PerFRACTION can be used generally to validate other forward‐projection transit dosimetry software. In addition, the measurement materials used are accessible to any physics department: solid water slabs and ionization chamber. Regarding the Monte Carlo simulations, PRIMO is free software and the PSFs are accessible from Varian. Therefore, the proposed methodology is easily adaptable to other systems.

## 5 CONCLUSION

We validated PerFRACTION calculated expected transit dose with PRIMO Monte Carlo software and ionization chamber measurements adapting the methodology of the MMPG5a report. The methodology presented can be applied to validate other forward‐projection transit dosimetry software.

## AUTHOR CONTRIBUTIONS

All authors contributed to the conception of the study, the data analysis, and the manuscript writing.

## CONFLICT OF INTEREST STATEMENT

The authors declare no conflict of interest.

## Supporting information

SUPPORTING INFORMATION
