# Supplementary material for: Validation of an in vivo transit dosimetry algorithm using Monte Carlo simulations and ionization chamber measurements
Source: J Appl Clin Med Phys. 2023 Oct 27;25(2):e14187. doi: 10.1002/acm2.14187 (PMC10860462; doi:10.1002/acm2.14187)
Supplement: Supplementary file 1 — SUPPORTING INFORMATION [file ACM2-25-e14187-s001.pdf]

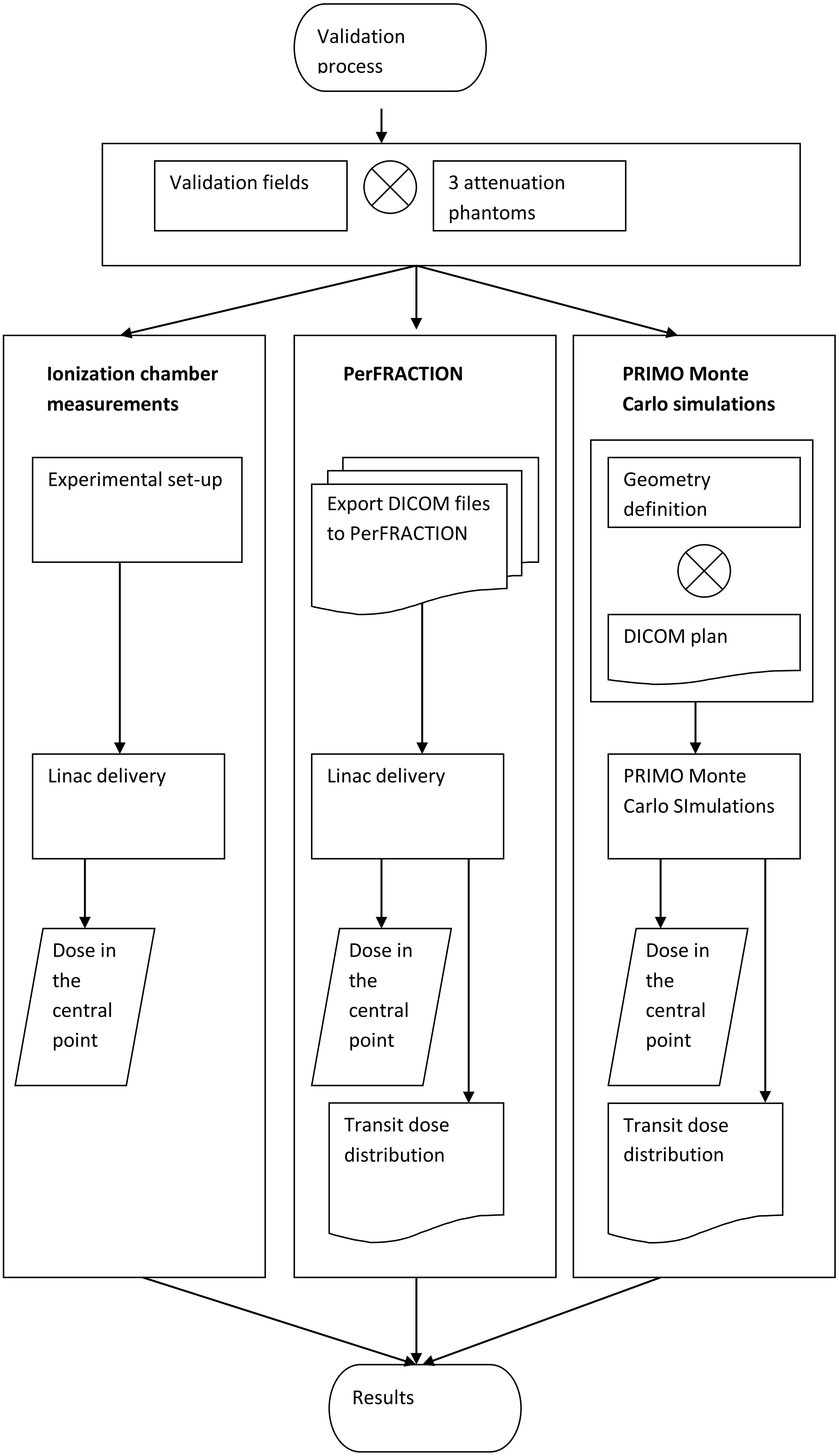
Validation process
Validation fields
3 attenuation phantoms
Ionization chamber measurements
Experimental set-up
Linac delivery
Dose in the central point
PerFRACTION
Export DICOM files to PerFRACTION
Linac delivery
Dose in the central point
Transit dose distribution
PRIMO Monte Carlo simulations
Geometry definition
DICOM plan
PRIMO Monte Carlo SImulations
Dose in the central point
Transit dose distribution
Results

Supplemntary material: flow chart describing the validation process.

Validation of an in-vivo transit dosimetry algorithm using Monte Carlo simulations and ionization chamber measurements

David Sánchez-Artuñedo, Savannah Pié-Padró, Marcelino Hermida-López, Maria Amor Duch-Guillén, Mercè Beltran-Vilagrasa
